# Severity-dependent functional connectome and the association with glucose metabolism in the sensorimotor cortex of Parkinson's disease

**DOI:** 10.3389/fnins.2023.1104886

**Published:** 2023-01-30

**Authors:** Zhenxiang Zang, Tianbin Song, Jiping Li, Binbin Nie, Shanshan Mei, Yuqing Zhang, Jie Lu

**Affiliations:** ^1^Department of Radiology and Nuclear Medicine, Xuanwu Hospital, Capital Medical University, Beijing, China; ^2^Beijing Key Laboratory of Magnetic Resonance Imaging and Brain Informatics, Beijing, China; ^3^Beijing Institute of Functional Neurosurgery, Xuanwu Hospital, Capital Medical University, Beijing, China; ^4^Beijing Engineering Research Center of Radiographic Techniques and Equipment, Institute of High Energy Physics, Chinese Academy of Sciences, Beijing, China; ^5^Department of Neurology, Xuanwu Hospital, Capital Medical University, Beijing, China

**Keywords:** Parkinson's disease, sensorimotor cortex, glucose metabolism, functional connectome, hybrid PET/MRI

## Abstract

Functional MRI studies have achieved promising outcomes in revealing abnormal functional connectivity in Parkinson's disease (PD). The primary sensorimotor area (PSMA) received a large amount of attention because it closely correlates with motor deficits. While functional connectivity represents signaling between PSMA and other brain regions, the metabolic mechanism behind PSMA connectivity has rarely been well established. By introducing hybrid PET/MRI scanning, the current study enrolled 33 advanced PD patients during medication-off condition and 25 age-and-sex-matched healthy controls (HCs), aiming to not only identify the abnormal functional connectome pattern of the PSMA, but also to simultaneously investigate how PSMA functional connectome correlates with glucose metabolism. We calculated degree centrality (DC) and the ratio of standard uptake value (SUVr) using resting state fMRI and ^18^F-FDG-PET data. A two-sample t-test revealed significantly decreased PSMA DC (P_FWE_ < 0.014) in PD patients. The PSMA DC also correlated negatively with H-Y stage (*P* = 0.031). We found a widespread reduction of H-Y stage associated (*P*-values < 0.041) functional connectivity between PSMA and the visual network, attention network, somatomotor network, limbic network, frontoparietal network as well as the default mode network. The PSMA DC correlated positively with FDG-uptake in the HCs (*P* = 0.039) but not in the PD patients (*P* > 0.44). In summary, we identified disease severity-dependent PSMA functional connectome which in addition uncoupled with glucose metabolism in PD patients. The current study highlighted the critical role of simultaneous PET/fMRI in revealing the functional-metabolic mechanism in the PSMA of PD patients.

## 1. Introduction

Despite the increasing number of studies that begin to shed light on cognitive impairment of Parkinson's disease (PD), the core symptom of this neurodegenerative disease has always been motor deficits such as resting tremor, akinesia or postural instability (Maiti et al., 2017). Being the cortical terminal of the cortical-basal ganglia-thalamus pathway (Calabresi et al., 2014) that has been disrupted by basal ganglia dysfunction in PD (DeLong, 1990; Dauer and Przedborski, 2003; McGregor and Nelson, 2019), the primary sensorimotor area (PSMA) has received enormous attention from various studies. Although promising outcomes regarding the PSMA functional connectivity and metabolic activity have been separately obtained by fMRI and ^18^F-fluorodeoxyglucose (FDG) PET studies (Ma et al., 2007; Wu et al., 2011; Thibes et al., 2017), yet how functional connectome of the PSMA associate with glucose metabolism in PD patients against the healthy population is still obscure.

Seed-based functional connectivity studies in relation to the PSMA were not convergent. While increased connectivity in the primary motor area (M1) was obtained Wu et al. (2009, 2011), Sharman et al. (2013) have revealed reduced connectivity within the sensorimotor cortex (Sharman et al., 2013) which is consistent with a longitudinal study that obtained reduced sensorimotor connectivity (Li et al., 2020). A potential reason for the diverse observations among the above-mentioned functional connectivity studies is the biased connectivity second to seed selection. Comparably, data-driven approaches like the degree centrality (DC) could assess the functional connectivity strength on a voxel-wise manner which allows researchers to investigate the functional connectome of the brain without prior selection of seed (Zuo et al., 2011). The DC approach has been successively applied in PD studies and researchers have consistently found reduced connectivity strength in the PSMA (Zhong et al., 2019; Guo et al., 2020). A thorough examination of the PSMA DC and its association with glucose metabolism may deepen the understanding of the functional-metabolic binding mechanism in PD patients.

Glucose metabolism is the physiological basis of the organization of functional networks as the majority of the energy consumption is dedicated to neural communication across species (Hyder et al., 2013). ^18^F-FDG-PET studies have reliably shown abnormal glucose metabolism in PD patients, including increased FDG-uptake in the cortical-basal ganglia-thalamus-cortical loop and reduction of FDG-uptake in the visual area and the frontoparietal networks (Schindlbeck and Eidelberg, 2018). Therefore, glucose metabolism plays both physiologically and pathologically a critical role in the architecture of system-level networks in PD patients. Recent studies applied multi-neuroimaging modality approach to study the inter-relationship among the dopamine impairment, abnormal glucose metabolism and functional network neurodegeneration in PD patients (Ruppert et al., 2020, 2021). However, the metabolic and functional data of these studies were acquired separately on PET and MRI scanners, limiting the strength of functional-metabolic investigation.

While the functional connectome described by the DC approach reflects how PSMA is functionally associated with the rest of the brain, the association with the metabolic basis, as measured via FDG-uptake is poorly studied. One critical reason is the lack of simultaneous acquisition of both the functional as well as metabolic data, causing difficulty for the combination of the two phenotypes. Here, we applied hybrid PET/MRI to simultaneously measure functional connectivity and glucose metabolism of the PSMA. Our overall aim is to investigate the how functional-metabolic coupling of PSMA functional connectome and glucose metabolism could vary in PD patients. We hypothesize that the PSMA may show both impaired functional connectivity and glucose metabolism in PD, and that the correlation between the two phenotypes may also vary in the two groups.

## 2. Materials and methods

### 2.1. Subjects

The study protocol was approved by the ethics committee of Xuanwu Hospital. After providing written informed consent, simultaneous PET/fMRI data were collected from 42 PD patients and age-and-sex balanced 25 HCs. Our data were previously reported (Zang et al., 2022a,b) where we investigated basal ganglia functional-metabolic features. Here, we instead focus on the sensorimotor cortex. Our PD patients were diagnosed with the movement disorder (MDS)-PD criteria (Postuma et al., 2015, 2018). PD patients who were younger than 40 years old or older than 75 years old were excluded for age-balancing purposes with the HCs. All participants were right-handed and reported no history of head trauma, psychiatric disease and cerebral vascular disease. To reduce the influence of head motion on fMRI data, we excluded eight PD patients who exceeded 30% of time points that were larger than 0.5 mm frame-wise displacement during data acquisition. PD patients were instructed to not use dopaminergic medication for at least 12 hours before the scan. Detailed information on all our subjects was provided in Table 1.

**Table 1:** Demographic information of subjects.

	**Healthy controls**	**Parkinson's disease**	***P*** **-value**	**Statistics**
Number	25	33 (42 recruited)		
Sex (m/f)	8 / 17	12 / 21	0.73	χ^2^ = 0.12
Age (±SD)	60.00 ± 4.54	62.33 ± 6.50	0.13	*T =* −1.53
FD Power (±SD)	0.22 ± 0.09	0.21 ± 0.08	0.66	*T =* 0.44
H-Y Stage (±SD)		2.94 ± 0.77		
UPDRS III (±SD)		59.73 ± 15.24		
Disease Duration (±SD)		9.58 ± 4.10		
MMSE (±SD)		23.07 ± 3.66		
MoCA (±SD)		26.67± 2.97		
Dopamine equivalent (±SD)		879.88 ± 432.81 (mg/day)		

Mean ± Standard deviations are shown. FD Power, Power's Frame-wise displacement; H-Y Stage, Hoehn and Yahr Stage; UPDRS III, Unified Parkinson's Disease Rating Scale part III; MMSE, Mini-Mental State Examination; MoCA, Montreal Cognitive Assessment.

### 2.2. Hybrid PET/MR data acquisition

All patients were fasted for at least 6 hours before PET/MR examination. The injected dose of ^18^F-FDG was 3.7 MBq/kg, with a one-hour average duration between tracer injection and hybrid PET/MR scan. PET/MR scan was performed on a hybrid PET/MR system (uPMR790, UIH) with 3.0T MR and a 24-channel coil. PET acquisition was 10 min. We reconstructed PET data using time of fly (TOF) approach based on the following parameters: iterations = 4, subsets = 20, Gaussian filter = 3 mm, matrix size = 2562 × 56, thickness = 2.8 mm, field of view (FOV) = 300 mm × 300 mm, and voxel size = 2.4 mm × 2.4 mm × 2.8 mm.

Before resting-state fMRI acquisition, we instructed subjects to close their eyes, relax, and not engage in any particular mental activity during the scan. Acquisition parameters were as follow: TR = 2000 ms, TE = 30 ms, slice thickness = 3.5 mm, voxel size = 3.5 × 3.5 × 3.5 mm^3^, 0.7 mm slice gap, 31 slices, 230 × 230 mm FOV, volume = 230 and 90° flip angle. The high-resolution 3-dimensional T1-weighted images were acquired with the following parameters: TR = 7.9 ms, TE = 3.8 ms, 176 slices, FOV = 256 × 256 mm, and 1 mm^3^ spatial resolution.

### 2.3. PET images processing

PET images were processed in SPM12. PET images were firstly co-registered to T1 images and then spatially normalized to the standard MNI template. An 8-mm full width at half maximum (FWHM) Gaussian kernel was used for spatial smoothing.

An iterative data-driven approach (Nie et al., 2018) for the ratio of standard uptake value (SUVr) was applied: (1) initial reference was defined as the whole brain, named “Ref0”; (2) the mean value of “Ref0” was used as global confounds for a voxel-wise two-sample t-test w between the preprocessed images of PD patients and HC; (3) The significant regions defined by the two-sample t-test was defined as “SigRegion” based on a P_uncorrected_ < 0.05 threshold; (4) create a new reference region “Ref1” by excluding “SigRegion” from “Ref0”; 5) use “Ref1” as the new global confound, and repeat steps 2-5 until the residual deviation between the “Ref1” and “Ref0” was reduced by less than 5%; (6) The latest “Ref1” was accepted as the data-driven unbiased reference region.

One patient was excluded due to enormous imaging artifacts, resulting in 58 subjects in total for further analyses (25 HC, 33 PD).

### 2.4. fMRI data processing

Image processing followed the standard routine in Statistical Parametric Mapping software (SPM12, https://www.fil.ion.ucl.ac.uk/spm/software/spm12/): (1) realigned for head motion correction; (2) slice timing (3) co-registering to the high-resolution 3D T1 images; (4) segmentation, and (5) spatial normalization of functional images via T1 images (resampled to 3 × 3 × 3 mm^3^). An 8-mm FWHM Gaussian kernel was applied for spatial smoothing. After spatial smoothing, we further regressed out the time series of white matter (WM 99% probability SPM map), cerebrospinal fluid (CSF 90% probability SPM map) (Zang et al., 2018), global mean time course, six head motion parameters from the realignment step, and the frame-wise displacement (FD) (Power et al., 2012). As mentioned above, we excluded subjects with over 30% time points that exceeded 0.5-mm FD. A 0.01-0.1 Hz band pass filtering was applied.

### 2.5. PSMA functional connectome

Firstly, we calculated voxel-wise binary degree centrality (DC) (Zuo et al., 2011). Voxel-by-voxel connections with *r* > 0.2 were counted as 1 while connections with *r* ≤ 0.2 were counted as 0. Each voxel's value on the resulting DC map indicates the number of voxels in the brain surpassed the 0.2 threshold (Wang et al., 2014; Takeuchi et al., 2015). For normalization purpose, the resulting DC map was normalized by dividing the individual's mean DC value across the brain.

Since we specifically focus on the functional connectome and the association with glucose metabolism in the PSMA, we applied the bilateral precentral gyrus and postcentral gyrus from the AAL template (Tzourio-Mazoyer et al., 2002) for group analyses. We applied a two-sample *t*-test with age, sex and FD as covariate of non-interests to localize the PSMA cluster that showed significant DC difference between PD and HCs. Clusters were defined using the following criteria: 1) peak voxel surviving family-wise error (FWE) correction (i.e., P_FWE_ < 0.05) within the PSMA mask and 2) cluster size > 10 voxels (i.e., 270 mm^3^) with P_uncorrected_ < 0.001 threshold. PSMA DC represents the functional connectome between PSMA and the rest of the brain.

To further analyze which network contributes to the difference of PSMA DC between PD patients and HCs, we decomposed the PSMA connection with other voxels in the brain into nine networks and analyzed the PSMA connection in each network. In detail, we calculated the ratio of voxels with *r* > 0.2 coefficient against the total number of voxels within each network as representation of network-specific DC linked to PSMA (PSMA DC ratio). The nine brain networks contained Yeo's seven networks (Yeo et al., 2011), the subcortical network as well as the cerebellum. We concatenated the thalamus, caudate, putamen, and globous pallidus for the construction of the subcortical network and all 26 subdivisions of cerebellar areas for the cerebellum from the AAL template.

### 2.6. Correlation analyses

We extracted the mean SUVr value from the significant PSMA cluster as the representation of FDG-uptake. Spearman's correlation was applied to calculate the association between PSMA functional connectome and FDG-uptake, as well as the clinical measurements (UPDRS III score, H-Y stage and disease duration). *P* < 0.05 was defined as the threshold for significance.

## 3. Results

### 3.1. Clinical and demographic data

The clinical and demographic data are summarized in Table 1. Thirty-three PD patients and 25 age (*P* = 0.13) and sex (*P* = 0.73) matched HCs were used for statistical analyses. PD patients exhibited balanced FD during fMRI scan (*P* = 0.66) compared to the HCs.

### 3.2. Reduced degree centrality in the PSMA

**Figure 1:**
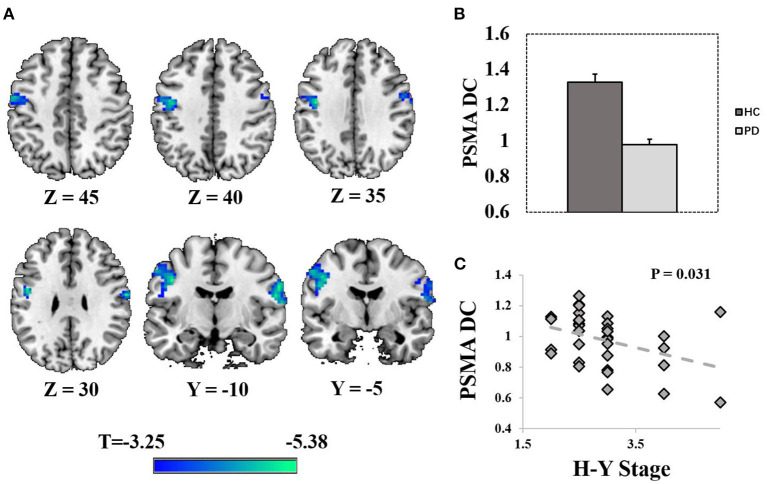
Display of significantly reduced degree centrality in the PSMA. **(A)** Shows the spatial distribution of the PSMA cluster (P_unccorrected_ < 0.001). **(B)** Shows the bar plots of normalized DC from the bilateral PSMA cluster. **(C)** Shows the correlation between normalized DC and the H-Y stage in PD patients. Error bars represent standard errors.

Compared with HCs, PD patients exhibited reduced DC in the PSMA (right PSMA: peak *T* = 5.38, P_FWE_ = 0.003, MNI = [63 −9 30], cluster size = 103 voxels at P_uncorrected_ < 0.001; left PSMA: peak *T* = 4.97, P_FWE_ = 0.014, MNI = [– 42 −9 36], cluster size = 145 voxels at P_uncorrected_ < 0.001; Figures 1A, B). Age, sex and FD were controlled as covariate of non-interests. *Post-hoc* analysis revealed a significant negative correlation between PSMA DC and the H-Y stage in the PD patients (rho = −0.38, *P* = 0.031, Figure 1C). No significant correlation was found between the PSMA DC and other clinical scores (UPDRS III, Disease duration, Mini-Mental State Examination, Montreal Cognitive Assessment *P*-values > 0.18).

### 3.3. PSMA DC ratio distribute differently in PD patients

**Figure 2:**
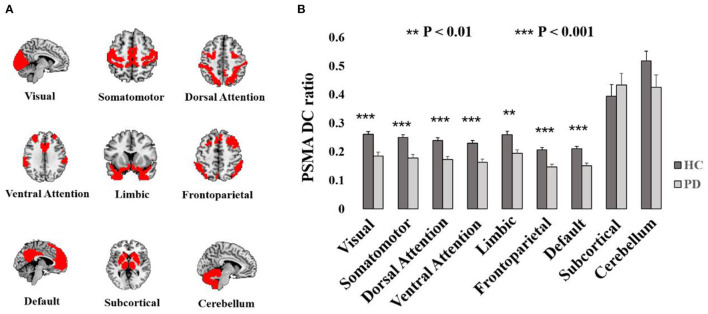
Display of nine networks and the group difference of PSMA DC ratio with the networks. **(A)** Illustrates the spatial pattern of Yeo's seven networks and the subcortical network as well as the cerebellum. **(B)** Shows the bar plots of PSMA DC ratio differences. Significant reduced PSMA DC ratio could be obtained in Yeo's seven networks (*P*-values < 0.01). Error bars represent standard errors.

Reduced PSMA DC in the PD patients and HCs distributed widely in the brain (P_uncorrected_ < 0.001, Supplementary Figure S1). The ratio of PSMA DC with r > 0.2 against the each of the nine network were significantly decreased in PD patients in the visual network (*T* = −4.19, *P* < 0.001), somatomotor network (*T* = −4.44, *P* < 0.001), dorsal attention network (*T* = −4.28, *P* < 0.001), ventral attention network (*T* = −4.55, *P* < 0.001), limbic network (*T* = −3.65, *P* = 0.001), frontoparietal network (*T* = −4.47, *P* < 0.001) and the default mode network (*T* = −4.42, *P* < 0.001). However, there was no group difference of the PSMA DC ratio in the subcortical network (*P* > 0.49) and the cerebellum (*P* > 0.12). Group differences of PSMA DC in the nine networks are shown in Figure 2.

In addition, we found significant negative correlations between H-Y stage and PSMA DC ratio in the visual network (rho = −0.47, *P* = 0.006), somatomotor network (rho = −0.50, *P* = 0.003), dorsal attention network (rho = −0.46, *P* = 0.007), ventral attention network (rho = −0.51, *P* = 0.003), limbic network (rho = −0.46, *P* = 0.007), frontoparietal network (rho = −0.48, *P* = 0.005), default mode network (rho = −0.46, *P* = 0.006) and the subcortical network (rho = −0.36, *P* = 0.042). Disease duration was significantly negatively correlated with PSMA DC ratio in the somatomotor network (rho = −0.39, *P* = 0.024), dorsal attention network (rho = −0.37, *P* = 0.035), ventral attention network (rho = −0.39, *P* = 0.025), frontoparietal network (rho = −0.35, *P* = 0.045) and the default mode network (rho = −0.36, *P* = 0.041). Although UPDRS III score was positively correlated with H-Y stage (*P* = 0.005), it did not correlate with PSMA DC ratio in any of the nine networks (P values > 0.5). Detailed correlation results between PSMA DC ratio and the clinical measurements are shown in Table 2.

**Table 2:** Spearman's correlation (rho) between clinical measurements and PSMA FCS with brain networks.

	**Visual**	**S.Motor**	**D. Attention**	**V. Attention**	**Limbic**	**FP**	**Default**	**Subcortical**	**Cerebellum**
UPDRS III	−0.088	−0.114	−0.117	−0.113	−0.067	−0.106	−0.087	−0.091	−0.036
H-Y Stage	−0.469^**^	−0.503^**^	−0.464^**^	−0.505^**^	−0.460^**^	−0.478^**^	−0.460^**^	−0.356^*^	−0.298
Duration	−0.324	−0.393^*^	−0.367^*^	−0.390^*^	−0.331	−0.351^*^	−0.358^*^	−0.213	−0.142

S.Motor, Somatomotor; D. Attention, Dorsal Attention; V. Attention, Ventral Attention; FP, Front parietal;

* P < 0.05;

** P < 0.01.

### 3.4. PSMA DC associate differently with FDG-uptake in PD patients against HCs

**Figure 3:**
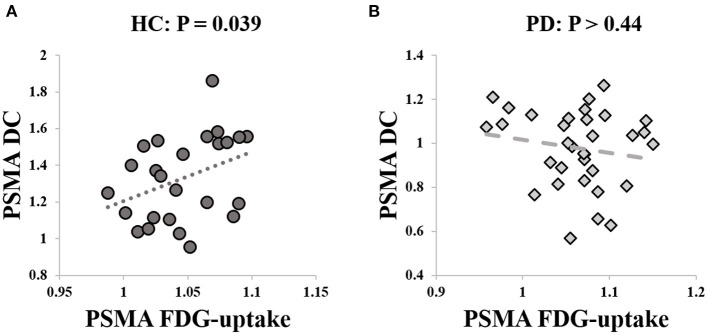
Correlation between PSMA DC and FDG-uptake. **(A)** Significant correlation was identified between PSMA DC and FDG-uptake in the HCs (rho = 0.42, *P* = 0.039). **(B)** The correlation between PSMA DC and FDG-uptake was not significant in PD patients (*P* > 0.44).

We extracted the mean SUVr value from the PSMA cluster. *Post-hoc* analysis revealed no significant group difference between PD and HCs (*P* > 0.14) with age and sex as covariate of non-interests. There was a significant positive correlation between the PSMA DC and SUVr value in the HCs (rho = 0.42, *P* = 0.039, Figure 3A), but not in the PD patients (*P* > 0.44, Figure 3B).

We performed two-sample t-test on the PET data and found increased FDG-uptake in different clusters within the PSMA (P_uncorrected_ < 0.001, Supplementary Figure S2). However, there was no spatial overlap between the increased FDG-uptake cluster and the reduced PSMA DC cluster. The SUVr value extracted from the P_uncorrected_ < 0.001 cluster in PSMA did not correlate with PSMA DC in any groups (*P*-values > 0.8), nor did it correlate with any clinical measurements (*P*-values > 0.15).

## 4. Discussion

In the current simultaneous PET/fMRI study, we investigated the functional connectome and the association with glucose metabolism in the primary sensorimotor area (PSMA) in PD patients. Our main results were: (1) significant reduced PSMA degree centrality (DC) in PD patients, which was also correlated with H-Y stage; (2) the PSMA degree centrality reduction in PD could be attributed to reduced DC ratio in the visual, attention, somatomotor, limbic, frontoparietal and default mode network and (3) the significant correlation between PSMA DC and glucose metabolism in the healthy population disappeared in PD patients. The main results will be discussed in the following paragraphs.

Degree centrality (DC) measures the number of connection linking to a node in the brain network. Here, we found reduced DC in the bilateral PSMA, meaning that the degree of functional strength decreased in PD patients. Similar observation was reported in a recently published meta-analysis study that cognitively normal PD patients showed reduced functional connectivity in the precentral gyrus (Wolters et al., 2019). In the current study, we also detected significant negative correlation between the PSMA DC and H-Y stage where severer PD patients showed severer loss-of-connection in PSMA. In addition, the location of significant PSMA DC reduction overlapped with pathological lesions corresponding to Braak's stage 5 and 6 (Braak et al., 2003), supporting the idea that PSMA DC reduction is a disease-severity functional feature.

By decomposing the functional connectome of PSMA into nine brain networks, we found significantly reduced connection between PSMA and visual, attention, somatomotor, frontoparietal, limbic and the default mode network. Further, connections between PSMA and these networks showed unanimously negative associations with H-Y stage and disease duration, suggesting a widespread disease severity-dependent pattern of PSMA functional connectome in PD patients. Loss-of-connection effect in the PSMA was reproducible in PD patients as similar observation was reported in previous studies (Guo et al., 2020; Suo et al., 2022). Further, PSMA DC could be significantly modulated by the deep brain stimulation in the subthalamic nucleus and internal globus pallidus, and the magnitude of PSMA DC alteration between on-off conditions was significantly correlated with motor behavior improvement (Zhang et al., 2021). Interestingly, the dysconnectivity of PSMA with the default mode network and the visual network may be associated with cognitive decline and visual hallucination (Tessitore et al., 2012; Zarkali et al., 2020), suggesting that the imbalanced network coupling of both motor and non-motor aspect that was influenced by the pathophysiology of parkinsonism. In addition, the reduced connectivity of the frontalparietal network may contribute to the lack of capability task-set maintenance in PD patients (Tinaz et al., 2016), as the frontal-parietal network was key to cognitive control and motor execution (Husárová et al., 2013). Together, the reduced functional connectivity strength of the PSMA in PD patients may denote an impairment of coordination within the large-scale network.

The network functional connectome exhibited only mediocre reliability (Noble et al., 2017) and a proportion of the reason being the variability of brain functions. Therefore, simultaneous PET/fMRI data acquisition is the base of capturing precise functional-metabolic coupling in PD patients. Comparable to previous study (Wu et al., 2013; Matthews et al., 2018), significant increased FDG-uptake in the PSMA was obtained. However, the cluster showing increased FDG-uptake located in adjacent to the paracentral lobule, which did not overlap with the cluster showing PSMA DC reduction. That being said, we did not obtain significant difference of FDG-uptake in the cluster showing the most significant PSMA DC reduction. Although the FDG-uptake in the PSMA DC cluster did not differ between PD patients and healthy populations, association between FDG-uptake and PSMA DC exhibited differently. In healthy population, a significant positive correlation between FDG-uptake and PSMA DC was obtained, indicating a coupling effect between PSMA functional signaling and energy consumption (Attwell and Laughlin, 2001; Harris et al., 2012). However, this coupling effect was disrupted in PD patients as the significant correlation between FDG-uptake and PSMA DC disappeared.

There are several limitations in the current study. Firstly, we recruited moderate to severe patients which limit the capability to explore how PSMA connectome and glucose metabolism distributed in mild even de novo PD patients. Secondly, although we found different correlations between PSMA DC and FDG-uptake between HC and PD, the clusters showing altered FDG-uptake distributed at a more superior level which is adjacent to the paracentral lobule. Therefore, we cannot fully exclude that the lack of correlation in the PD group was partially due to the location of clusters showing increased FDG-uptake.

## Conclusion

In conclusion, the current study is, to the best of our knowledge, the first to utilize simultaneous PET/fMRI data acquisition protocol to investigate the disruption of functional connectome and the association with glucose metabolism in the primary sensorimotor area of PD patients. We identified an uncoupling effect between glucose metabolism and functional connectome feature in PD patients. The current study not only provided metabolic basis for explaining the impairment of functional connectome in the primary sensorimotor area in PD patients, but also highlighted the critical role of hybrid PET/MRI scanner in revealing functional-metabolic mechanism of this neurodegeneration disease.

## Data availability statement

The original contributions presented in the study are included in the article/Supplementary material, further inquiries can be directed to the corresponding author.

## Ethics statement

The studies involving human participants were reviewed and approved by the Ethics Committee of Xuanwu Hospital. The patients/participants provided their written informed consent to participate in this study.

## Author contributions

ZZ: manuscript preparation, study concept, design, analysis, and interpretation of data. TS: manuscript revision and data acquisition. JLi and SM: data acquisition and clinical assessment. BN: data analysis. YZ: data acquisition, manuscript revision, and critical review. JLu: study concept, manuscript revision, and critical review. All authors contributed to the article and approved the submitted version.
