# Supplementary material for: Severity-dependent functional connectome and the association with glucose metabolism in the sensorimotor cortex of Parkinson's disease
Source: Front Neurosci. 2023 Jan 30;17:1104886. doi: 10.3389/fnins.2023.1104886 (PMC9922997; doi:10.3389/fnins.2023.1104886)

**Severity-Dependent Functional Connectome and the Association with Glucose Metabolism in the Sensorimotor Cortex of Parkinson’s Disease**

**Supplementary Information**

**Figure S1. Results of two sample T-test showing decreased PSMA connectivity in PD patients (P < 0.001, uncorrected).**


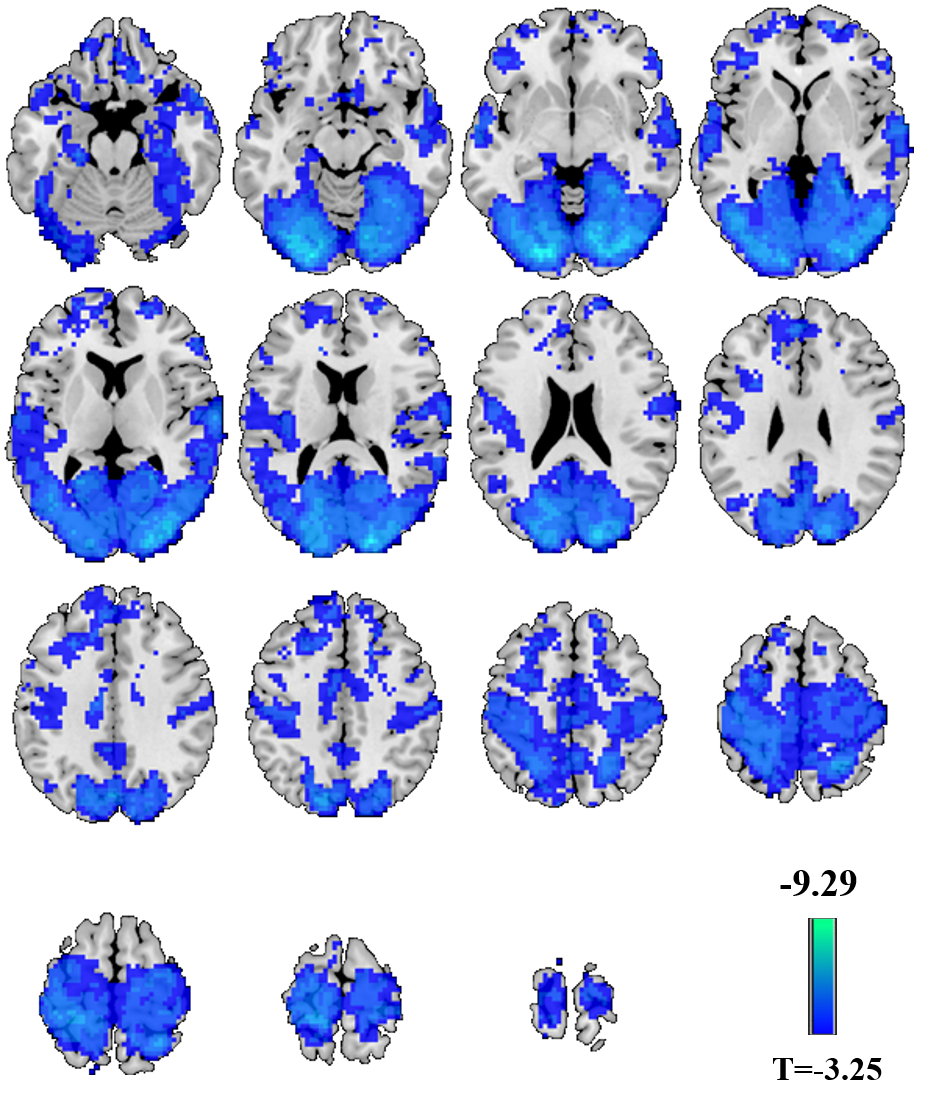


**Figure S3. Results of two sample T-test showing altered FDG-uptake in PD patients (P < 0.001, uncorrected).**


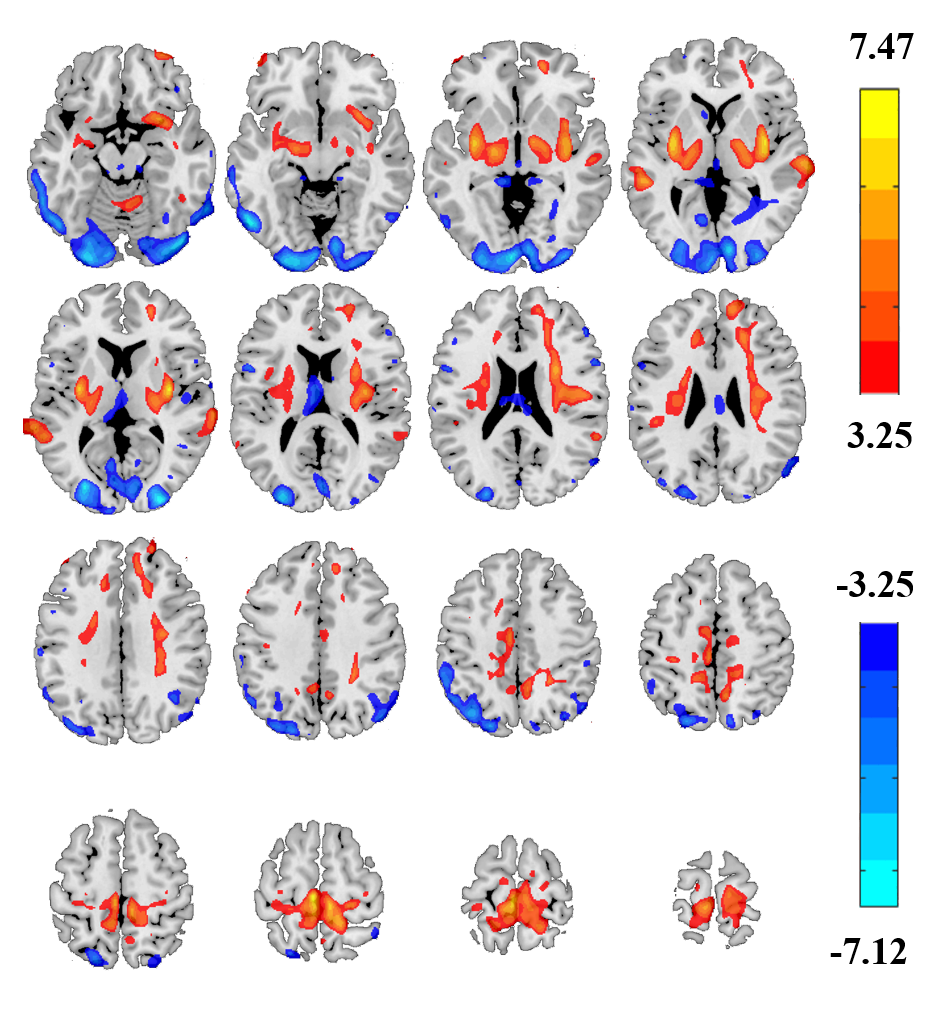

Supplement: Supplementary file 1 [file Data_Sheet_1.docx]
